# Endothelial Sirtuins and Mitochondrial Function Are Associated With Testosterone Status: Implications for Accelerated Vascular Aging in Middle‐Age and Older Men With Low Testosterone

**DOI:** 10.1111/acel.70457

**Published:** 2026-04-15

**Authors:** Branden L. Nguyen, Mackenzie N. Kehmeier, Matthew C. Babcock, Lyndsey E. DuBose, Kerry L. Hildreth, Brian L. Stauffer, Ryan Rosenberry, Amy C. Keller, Kira Steinke, Kaleb Miles, Lucas Guerrero, Wendy M. Kohrt, Jane Reusch, Zachary S. Clayton, Kerrie L. Moreau

**Affiliations:** ^1^ School of Medicine, Division of Geriatric Medicine University of Colorado Anschutz Medical Campus Aurora Colorado USA; ^2^ School of Medicine, Division of Cardiology University of Colorado Anschutz Medical Campus Aurora Colorado USA; ^3^ Division of Cardiology, Denver Health Medical Center Denver Colorado USA; ^4^ School of Medicine, Division of Endocrinology, Metabolism, and Diabetes University of Colorado Anschutz Medical Campus Aurora Colorado USA; ^5^ VA Eastern Colorado Health Care System Aurora Colorado USA; ^6^ Eastern Colorado Geriatrics Research, Education, and Clinical Center, VA Eastern Colorado Health Care System Aurora Colorado USA

**Keywords:** aging, endothelial dysfunction, hypogonadism, mitochondria, sex hormones

## Abstract

Middle‐aged/older (MA/O) men with low testosterone have greater oxidative stress‐mediated vascular endothelial dysfunction, a major risk factor for cardiovascular disease (CVD). Testosterone deficiency impairs mitochondria, a source and target of oxidative stress. Whether the greater vascular endothelial dysfunction in MA/O men with low testosterone is related to mitochondrial dysfunction is unknown. This cross‐sectional study measured mitochondrial respiration in peripheral blood mononuclear cells (**PBMCs**), and regulators of mitochondrial function (i.e., sirtuins [**SIRTs**]), and oxidant burden in vascular endothelial cells from (1) young adult men with normal testosterone (18–40 years; serum testosterone ≥ 13.9 nmol/L [400 ng/dL]; *n* = 23); (2) MA/O men with normal testosterone (50–75 years; serum testosterone ≥ 13.9 nmol/L [400 ng/dL]; *n* = 57), and (3) MA/O men with low testosterone (50–75 years; serum testosterone < 10.4 nmol/L [300 ng/dL]; *n* = 21). PBMCs from MA/O men with low testosterone had reduced carbohydrate (2.96 ± 0.65 vs. 6.85 ± 0.77 pmol/s·million cells; *p* = 0.001) and lipid‐supported (4.10 ± 0.67 vs. 6.23 ± 0.69 pmol/s·million cells; *p* = 0.047) state 2 respiration compared to young men, and lower carbohydrate‐supported uncoupled respiration than age‐matched men with normal testosterone (17.77 ± 2.91 vs. 24.9 ± 1.93 pmol/s·million cells; *p* = 0.046). SIRT3 arterial (0.64 ± 0.04 vs. 0.99 ± 0.08 FU; *p* = 0.003) and venous (0.61 ± 0.03 vs. 0.92 ± 0.07 FU; *p* = 0.003) expression was lower in endothelial cells from MA/O men with low testosterone compared to age‐matched men with normal testosterone. This study highlights the potential role of mitochondrial respiration and regulation in accelerated vascular aging in hypogonadal MA/O men. Importantly, these findings provide promising evidence for clinical therapeutic interventions to target mitochondrial health and SIRT3 to mitigate accelerated vascular aging in hypogonadal MA/O men.

## Graphical abstract

Middle‐aged/older (MA/O) men with low testosterone have greater age‐related vascular endothelial dysfunction than MA/O with normal testosterone. This study highlights the potential role of mitochondrial dysfunction and reduced sirtuin 3, a key modulator of mitochondrial function and oxidative stress, in accelerated vascular aging in MA/O men with low testosterone. These results provide promising evidence for clinical therapeutic interventions that target SIRT3 and mitochondrial health to mitigate accelerated vascular aging in MA/O men with low testosterone. Created in Biorender. https://BioRender.com/jbn0jdm. Moreau* (2026).

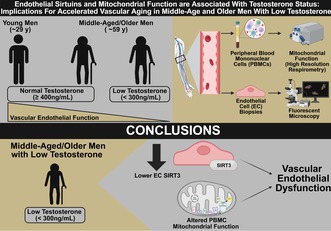

## 1 Introduction

Aging is associated with a gradual decline in serum testosterone levels at a rate of 1%–2% per year after the third decade of life, with ~20% of 60‐year‐old and ~50% of 80‐year‐old men having total testosterone levels below the normal range for young men (Harman et al. 2001). Low testosterone levels nearly double the risk of mortality when compared with normal levels of testosterone (Shores et al. 2006). The increased mortality risk is largely driven by the development of cardiovascular disease (CVD) (Hyde et al. 2012; Shores et al. 2014). Thus, there is a need to elucidate the pathobiology underlying low testosterone and CVD risk and mortality in hypogonadal aging men.

Vascular aging, featuring endothelial dysfunction mediated by oxidative stress (i.e., excessive reactive oxygen species [ROS]) and inflammation (A. J. Donato et al. 2007; Eskurza et al. 2004; Taddei et al. 2001; Moreau et al. 2013; Walker et al. 2014), is a major risk factor for the development of CVD. Previously, our group reported that middle‐aged/older (MA/O) men with low testosterone have greater age‐associated vascular endothelial dysfunction than age‐matched men with normal testosterone, related in part to greater systemic oxidative stress and inflammation (M. C. Babcock et al. 2022). The mechanisms underlying the vascular endothelial dysfunction associated with oxidative stress and inflammation in MA/O men with low testosterone are not completely understood but may be related to dysfunctional mitochondria. Mitochondria play a crucial role in maintaining cellular homeostasis and vascular function, but become increasingly damaged with aging, leading to increased production of ROS (Mikhed et al. 2015). It is well established that mitochondria are the largest generators of ROS, particularly at complex I and III, where excessive ROS production can amplify oxidative damage (Chen et al. 2003). Moreover, limited substrate flexibility (the capacity to switch between multiple energy sources) can further contribute to excessive ROS production from mitochondria (Muoio 2014).

Major regulators of mitochondrial ROS, antioxidant defenses, and metabolic flexibility are sirtuins 1 and 3 (SIRT1 and SIRT3), which are proteins that act as NAD+‐dependent deacetylases. SIRT1 is primarily localized in the nucleus or cytoplasm and supports mitochondrial function via effects on mitochondrial biogenesis, mitochondrial dynamics, metabolism, and stress response (Li et al. 2025). SIRT3 is localized in the mitochondria, directly deacetylating and activating enzymes involved in the tricarboxylic acid (TCA) cycle, fatty acid oxidation, and antioxidant defense (i.e., manganese superoxide dismutase [MnSOD]) (Trinh et al. 2024). Preclinical studies suggest testosterone deficiency impairs mitochondrial function and testosterone treatment can increase SIRT1 endothelial expression, and improve mitochondrial biogenesis and oxidative phosphorylation in other tissues (F. Wang et al. 2015; Liu et al. 2019; Rossetti and Gordon 2017; Tsuchiya et al. 2020). However, whether the apparent acceleration in vascular aging in MA/O men with low testosterone is related to mitochondrial dysfunction is unknown.

Building on our previous observations (M. C. Babcock et al. 2022), we sought to further elucidate the cellular/molecular mechanisms by which low testosterone influences age‐associated vascular endothelial function. Thus, we tested the integrative hypothesis that the greater age‐related vascular endothelial dysfunction in MA/O men with low serum testosterone is associated with greater intracellular impairments in mitochondrial function and oxidant stress, and lower SIRT1 and SIRT3 in vascular endothelial cells compared to MA/O men with normal testosterone. To test this hypothesis, we measured vascular endothelial function (i.e., brachial artery flow‐mediated dilation, FMD) and markers of mitochondrial health in peripheral blood mononuclear cells (PBMCs) and in primary vascular endothelial cells in young and MA/O men with normal testosterone, and MA/O men with low testosterone.

## 2 Methods

### 2.1 Study Design

The study design was previously published (M. C. Babcock et al. 2022). Briefly, this cross‐sectional study was part of a registered clinical trial (ClincialTrials.gov identifier NCT02758431). The Colorado Multiple Institution Review Board approved all study protocols and procedures and conformed to the provisions of the Declaration of Helsinki. All participants provided written and verbal consent prior to participating. All study visits and measurements were performed at the Colorado Clinical and Translational Sciences Institute (CCTSI) Clinical and Translational Research Center (CTRC) (M. C. Babcock et al. 2022).

### 2.2 Participant Characteristics

One hundred one men of all races and ethnic backgrounds aged 50–75 years for middle‐aged/older and 18–40 years (young, *n* = 23) were recruited from the Denver metropolitan area. Middle‐aged/older men were categorized into two groups: normal testosterone (serum T ≥ 13.9 nmol/L [400 ng/dL], *n* = 55) or low testosterone (serum T < 10.4 nmol/L [300 ng/dL], *n* = 23) at screening. All serum testosterone levels were measured in the morning under fasted conditions and confirmed on at least one other occasion, as previously described (M. C. Babcock et al. 2022). Inclusion criteria were: (1) no use of sex hormones for at least 1 year; (2) body mass index (BMI) < 40 kg/m^2^; (3) nonsmoker; (4) resting blood pressure < 160/90 mmHg; (5) nondiabetic and fasted plasma glucose < 7.0 mmol/L (126 mg/dL); (6) healthy and free from cardiovascular, cancer, renal, liver, or respiratory diseases as assessed by medical history, physical exam, standard blood chemistries (comprehensive metabolic panel, complete blood count, and thyroid stimulating hormone), and electrocardiography at rest and during a graded exercise treadmill test to fatigue; (7) sedentary or recreationally active (self‐report of < 3 days/week of vigorous aerobic exercise); (8) no use of medications that might influence cardiovascular function including antihypertensive and lipid‐lowering medications; and (9) no use of vitamin supplements or anti‐inflammatory medications, or willing to stop 1 month prior and throughout the study (M. C. Babcock et al. 2022).

### 2.3 Blood Sampling

Blood samples were collected under fasted conditions on the day of vascular testing, and all serum and plasma assays were performed at the CTRC Core Laboratory as previously described (M. C. Babcock et al. 2022). Briefly, glucose, insulin, total cholesterol (Roche Diagnostic Systems, Indianapolis, IN), and high‐density lipoprotein (HDL) cholesterol (Diagnostic Chemical Ltd., Oxford, CT) were determined in plasma using enzymatic/colorimetric methods. Enzyme‐linked immunosorbent plate assay (Alpco Diagnostics, Windham, NH) was used to measure oxidized low‐density lipoprotein (LDL). Serum was used to measure total testosterone, estradiol, sex hormone binding globulin (SHBG), follicle stimulating hormone (FSH), and luteinizing hormone (LH), by chemiluminescence using a Beckman Coulter Access II analyzer. Interleukin‐6 (IL‐6) was measured by enzyme‐linked immunoassay, and high‐sensitivity C‐reactive protein (CRP) was measured by the immunoturbidimetric method. Free testosterone was calculated for each participant from concentrations of serum testosterone, SHBG, and albumin using an online algorithm (www.issam.ch) using the Vermeulen equation (Vermeulen et al. 1999).

### 2.4 Macrovascular Endothelial Function

The procedure for assessing vascular endothelial dysfunction was described previously (M. C. Babcock et al. 2021). Briefly, a cuff was placed around the forearm, and images of brachial artery diameter and blood flow velocity were acquired ~3–6 cm above the antecubital fossa. The ultrasound probe was held in place with a stereotactic clamp to ensure the location of the arterial segment remained constant and the isonation angle was maintained at ≤ 60°. After obtaining concurrent measures of brachial artery diameter and blood flow velocity, forearm occlusion was produced by inflating the cuff to 250 mmHg, and occlusion was maintained for 5 min. Prior to the release of the cuff, Doppler blood flow velocity was acquired and recorded until ~15 s after rapid release of arterial occlusion. B‐mode ultrasound brachial artery diameter images were recorded continuously for 2 min. The dilation of the brachial artery in response to the stimulus of forearm ischemia is dependent on the release of vasodilators, predominantly nitric oxide (NO), from the vascular endothelium (Doshi et al. 2001).

### 2.5 Mitochondrial Respiration

PBMCs and endothelial cells primarily rely on glucose as a substrate for their energy source; however, mitochondria can also utilize lipids to produce ATP via beta‐oxidation (Zeng et al. 2018; Newsholme et al. 1985). The rationale for using PBMCs as a proxy for endothelial cells was two‐fold. First, limitations in cell quantity necessitated the use of PBMCs; and, second, PBMCs have been used successfully as surrogate markers of overall mitochondrial function in various diseases (Sharma et al. 2025) and have been implicated in CVD risk (DeConne et al. 2020; Sauer et al. 2022). PBMCs were isolated by gradient centrifugation. One 10 mL ethylenediaminetetraacetic acid (EDTA) vacutainer of blood was added to one tube of Dulbecco's phosphate‐buffered saline and carefully layered into a tube of Lymphocyte Separation Medium (LSM). LSM + blood mixture was centrifuged and spun (2800 rpm at 10°C for 40 min). Mitochondrial respiration was measured using Oroboros Oxygraph‐2k (O2k; Oroboros Instruments Corp., Innsbruck, Austria) on isolated PBMCs from participant blood samples. To prepare for this instrumentation, cells were resuspended in MiR05 respiration buffer (0.5 mM egtazic acid (EGTA), 3 mM magnesium chloride, 60 mM K‐lactobionate, 20 mM taurine, 10 mM potassium phosphate, 20 mM 4‐(2‐hydroxyethyl)‐1‐piperazineethanesulfonic acid (HEPES), 110 mM sucrose, 1 g/L fatty acid‐free bovine serum albumin) and PBMCs were added to the chamber and permeabilized with 3.5 μM digitonin. Substrates and inhibitors to mimic carbohydrate and lipid metabolism were added to assess respiration rates. Carbohydrate substrates were as follows: 5 mM pyruvate, 2 mM malate, 10 mM glutamate, four titrations of 0.5 mM adenosine diphosphate (ADP; final 2 mM), two titrations of 1 M succinate (final 6 mM), 2 μg/mL oligomycin, and 0.5 μM/step of carbonyl cyanide‐p‐trifluoromethoxyphenylhydrazone (FCCP). Lipid substrates: 0.025 mM octanoylcarnitine, 2 mM malate, four titrations of 0.5 mM ADP (final 2 mM), 10 μM glutamate, 6 mM succinate (one titration added together with glutamate, and one following glutamate), 2 μg/mL oligomycin, and 0.5 μM/step of FCCP. Cells were counted under a microscope using a hemocytometer after the experiment, and traces were normalized to cell count.

### 2.6 PBMC Protein Expression

After PBMCs were isolated, cells were stored in phosphate‐buffered saline at −80°C. When ready to process, cells were thawed on ice, spun at 4°C at 800 *g* for 10 min, and resuspended in mammalian lysis buffer (MPER with 150 mmol/L sodium chloride, 1 mmol/L of EDTA, 1 mmol/L EGTA, 5 mmol/L sodium pyrophosphate, 1 mmol/L sodium orthovanadate, 20 mmol/L sodium fluoride, 500 nmol/L okadaic acid, 1% protease inhibitor cocktail). Samples were then sonicated, and cells were centrifuged at 1000 *g* at 4°C for 10 min. The Bradford assay was used to measure the protein concentration of the cell lysate (supernatant). The WES (ProteinSimple, Minneapolis, MN, USA), a 12–230 kDa separation module (ProteinSimple SM‐W004), was used for proteins (Beta Actin 1:50, MnSOD 1:25, Mfn2 1:50, COXIV 1:50, DRP1 1:100). Anti‐Rabbit Detection Kits (ProteinSimple DM‐001) were used. Briefly, cell lysates, standards, and reagents were prepared per manufacturer instructions. Samples and standards were applied to the WES capillary plate and analyzed on the WES instrumentation per manufacturer instructions. Although multiple exposures were collected by the WES, optimization led to the use of the 5‐s exposures for all targets to avoid saturation. All data were normalized to β‐actin.

For Western blotting, PBMCs were isolated, processed, and assessed for protein content as described above. Protein samples (15 μg) in Laemmli sample buffer (LSB, boiled with 100 mmol L‐1 dithiothreitol [DTT]) were analyzed on precast sodium dodecyl sulfate‐polyacrylamide‐4‐15% (SDS) gels. Proteins were transferred to polyvinylidene difluoride (PVDF) membranes. Quantity One, Bio‐Rad, was used to evaluate protein loading. Blots were probed with a total oxphos complex mitochondrial antibody 1:500 (Total Oxphos Blue Native Antibody Cocktail MS603‐300, Abcam, previously MitoSciences) and left overnight at 4°C. Secondary antibody was applied following the primary antibody incubation (1:10,000 IRDye800CW, LicorBio), 1 h at room temperature. Total protein was measured using the activation of stain‐free gels (www.bio‐rad.com)for the ChemiDoc Imaging System (Bio‐Rad, Hercules, CA). Quantity One 1‐D Analysis software (Bio‐Rad, Hercules, CA) was used for analysis.

### 2.7 Vascular EC Protein Expression

A subset of men underwent harvesting of arterial (*n* = 71) and/or venous (*n* = 96) endothelial cells. The procedures used for the harvest and protein measurements of vascular endothelial cells have been previously described (Babcock et al. 2025; Donato et al. 2007; Silver et al. 2007; Silver et al. 2010). Endothelial cells were collected from the brachial artery and antecubital vein using sterile J‐wires (~4 cm beyond the tip of an 18‐gauge [for venous endothelial cells] or 3.0 French [for arterial endothelial cells] catheter) that were advanced and retracted. The wires were then transferred to a disassociation buffer and immediately washed and fixed with 3.7% formaldehyde and plated on poly‐L‐lysine coated slides (Sigma Chemical, St Louis Mo., USA). Slides were stored in −80°C freezer until they were removed for staining (Silver et al. 2010).

For immunofluorescent staining, cells were rehydrated with PBS and made permeable using 0.1% Triton X‐100 (Alfa Aesar, Ward Hill, MA). Nonspecific binding sites were blocked using 5% donkey serum (Jackson Immunoresearch, West Grove, PA, USA), and then arterial and venous endothelial cells were incubated for one of the following antibodies and their subsequent dilutions: sirtuin 1 (1:150), sirtuin 3 (1:250), magnesium superoxide dismutase (MnSOD; 1:645), and nitrotyrosine (1:300).

Slides were viewed using a fluorescence microscope (Eclipse 80i, Nikon, Melville, NY) and approximately 30 images were captured per slide (Photometrics CoolSNAPfx, Roper Scientific Inc., Tucson, AZ). Analysis methods were performed as previously described (Silver et al. 2010; Babcock et al. 2025). Briefly, endothelial cells were identified by von Willebrand factor, and nuclear integrity was confirmed by DAPI (4′,6′‐diamidino‐2‐phenylindole hydrochloride). Once endothelial cells with intact nuclei were identified, images were analyzed with NIS Elements Basic Research (version 4.20.02, Nikon, Melville, NY) to quantify the intensity of staining (average pixel intensity). Values are reported as ratios of endothelial cell protein expression/HUVEC. A single technician analyzed each batch of slides and was blinded to the participant's identity during staining and analysis.

### 2.8 Statistics

Normally distributed (Gaussian; Shapiro–Wilk test) participant characteristics were examined using one‐way ANOVA, with Tukey's post hoc analysis to examine differences between groups if data were normally distributed. If data were not normally distributed, a one‐way nonparametric test (Kruskal–Wallis) with Dunn's multiple comparisons was used. Relations between continuous variables were analyzed using Pearson correlations (if data were normally distributed) or Spearman correlations (if data were not normally distributed). Outliers were determined via Robust Regression and Outlier Removal (ROUT) method, which uses nonlinear regression to identify outliers from the dataset (Q was set at 1%). FMD was also examined using ANCOVA, and estimated means for FMD were calculated adjusting for BMI and SBP. Statistical analyses were completed using GraphPad Prism 10.4.0 and IBM SPSS 28.

## 3 Results

### 3.1 Demographics and Clinical Characteristics

The characteristics of the population are presented in Table 1. Middle‐aged/older men had higher systolic and diastolic blood pressure, body mass index, waist circumference and waist to hip ratio (WHR) compared with young men (all *p* < 0.001). WHR was higher in middle‐aged/older men with low testosterone compared to middle‐aged/older men with normal testosterone. By design, serum total testosterone and free testosterone were higher in young (total testosterone, *p* < 0.001; free testosterone, *p* < 0.001) and MA/O men with normal serum testosterone (total testosterone, *p* < 0.001; free testosterone, *p* < 0.001) compared with MA/O men with low testosterone. Middle‐aged/older men with normal testosterone had higher levels of estradiol (*p* = 0.03), SHBG (*p* < 0.001), white blood cell count (*p* < 0.001), and lower levels of insulin (*p* < 0.001) compared to MA/O with low testosterone. Both MA/O men with normal and low testosterone levels had higher total and LDL‐cholesterol, oxidized LDL, IL‐6 (all *p* < 0.001), triglycerides (*p* = 0.01), and CRP (*p* = 0.009) compared to young men. Brachial artery FMD was lower in MA/O men compared to young men (Table 1) but when adjusting for both BMI and SBP, FMD was lowest in the MA/O men with low testosterone compared with young and MA/O men with normal testosterone (*p* = 0.04; Supplemental Figure S1) consistent with our previous observation (Babcock et al. 2022).

**TABLE 1:** Participant characteristics.

Group	Young	Middle‐aged/older (normal testosterone)	Middle‐aged/older (low testosterone)	*p*
*N*	22	55	23	
Age (years)	29.5 ± 4.4	58.7 ± 6.1*	59.3 ± 7.3*	< 0.001
SBP (mmHg)	117 ± 9	125 ± 10*	129 ± 9*	< 0.001
DBP (mmHg)	73 ± 6	80 ± 7*	81 ± 7*	< 0.001
FMD (%)	7.34 (6.8–8.1)	4.79 (3.1–6.5)	3.7 (2.5–4.7)	< 0.001
Heart rate (bpm)	61 ± 10	60 ± 8	65 ± 13	0.19
Height (cm)	180.0 ± 6.5	179.5 ± 6.7	176.1 ± 7.7	0.14
Weight (kg)^a^	77.3 (68.8–82.5)	85.7 (78.5–97.0)*	94.1 (81.7–106.9)*	< 0.001
BMI (kg/m^2^)	23.8 ± 2.8	27.6 ± 4.0*	30.0 ± 4.8*	< 0.001
Waist circumference (cm)	85.5 ± 8.2	100.4 ± 9.7*	105.6 ± 12.8*	< 0.001
Hip circumference (cm)	96.6 ± 5.8	107.8 ± 7.8*	108.2 ± 12.8*	< 0.001
WHR	0.88 ± 0.10	0.93 ± 0.05*	0.98 ± 0.08* ^,^ **	< 0.001
Testosterone (ng/dL)^a^	514 (456–539)	481 (432–524)	266 (231–293)* ^,^ **	< 0.001
Free testosterone (ng/dL)^a^	11.8 (8.5–13.1)	8.5 (7.5–10.0)*	6.6 (5.3–8.1)*	< 0.001
Estradiol (pg/mL)^a^	32 (20–38)	43 (37–54)*	35 (26–46)**	0.003
SHBG (nmol/L)^a^	28 (21–36)	40 (34–49)*	25 (21–27)**	< 0.001
FSH (mIU/mL)	3.6 ± 1.9	5.7 ± 5.0	7.2 ± 9.5	0.094
LH (mIU/mL)^a^	3.0 (2.3–4.6)	3.1 (2.5–4.1)	2.9 (1.6–3.9)	0.98
Glucose (mg/dL)	87 ± 8	90 ± 8	94 ± 7*	0.014
Insulin (uIU/mL)^a^	3.0 (2.0–4.0)	3.0 (2.0–5.0)	6.0 (2.0–8.5)* ^,^ **	< 0.001
Total cholesterol (mg/dL)	154 ± 28	187 ± 37*	188 ± 27*	< 0.001
HDL‐cholesterol (mg/dL)^a^	45 (39–52)	46 (41–55)	42 (36–52)	0.269
LDL‐cholesterol (mg/dL)^a^	103 (82–126)	132 (112–150)*	147 (137–165)*	< 0.001
Oxidized LDL‐C (U/L)^a^	54 (44–86)	68 (60–77)	73 (61–82)	< 0.001
Triglycerides (mg/dL)^a^	76 (49–106)	84 (67–108)	129 (89–188)*	0.01
WBC	5.1 ± 1.3	4.9 ± 1.2	6.4 ± 2.2* ^,^ **	< 0.001
IL‐6 (pg/mL)^a^	0.8 (0.61–1.0)	1.5 (1.1–2.0)*	2.0 (1.4–3.0)*	< 0.001
CRP (mg/L)^a^	0.4 (0.27–0.6)	1.1 (0.55–2.5)*	1.6 (1.2–4.5)*	0.009

*Note:* Data were examined using a one‐way ANOVA, displayed as mean ± SD except in the case of non‐normally distributed data.

Abbreviations: BMI, body mass index; CRP, C‐reactive protein; DBP, diastolic blood pressure; HDL, high‐density lipoprotein; IL‐6, interleukin‐6; LDL, low‐density lipoprotein; SBP, systolic blood pressure; SHBG, sex hormone binding globulin; WBC, white blood cell count; WHR, waist‐to‐hip ratio.

^a^ Non‐normally distributed data, examine using Kruskal–Wallis and are displayed as median ± interquartile range.

* *p* < 0.05 versus young.

** *p* < 0.05 versus middle‐aged/older normal testosterone.

### 3.2 The Effects of Age and Gonadal Status on Mitochondrial Function and Mitochondrial Complex Abundance

**FIGURE 1:**
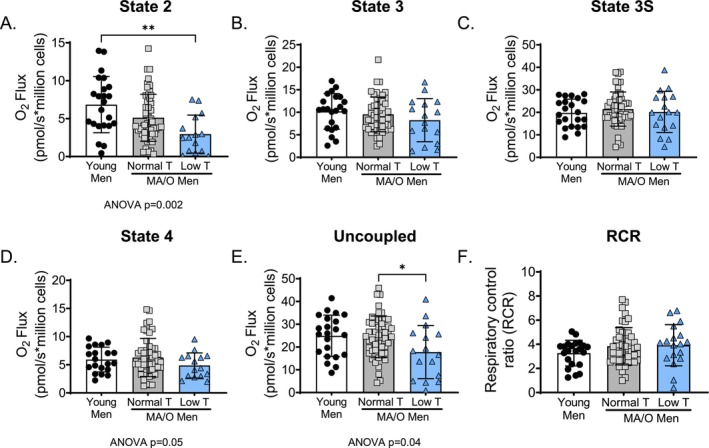
Carbohydrate supported mitochondrial function in human peripheral blood mononuclear cells (PBMCs). (A) State 2 (leak state), (B) State 3 (complex I [CI] coupled), (C) State 3S [CI+(complex II] CII coupled), (D) State 4 (leak respiration), (E) Uncoupled respiration (maximal respiration), and (F) Respiratory control ratio (RCR; State 3S/State 4), in young and middle aged/older (MA/O) males with normal testosterone and low testosterone. Respiration rate normalized to cell count. *p* values represent a one‐way ANOVA, with Tukey post hoc or Kruskal–Wallis with Dunn's multiple comparison if data were not normally distributed. *p < 0.05, **p < 0.01 (*n* = 15–33/group). Data are mean ± SD.

Carbohydrate‐supported state 2 respiration was lower in MA/O men with low testosterone compared to young men (*p* = 0.001, Figure 1A). There was a trend for carbohydrate‐supported state 2 respiration to be lower in MA/O men with low testosterone compared to MA/O men with normal testosterone (*p* = 0.058; Figure 1A). There was also a trend for uncoupled maximal respiration to be lower in MA/O men with low testosterone compared to young men (*p* = 0.06; Figure 1E). Uncoupled maximal respiration was lower in MA/O men with low testosterone compared to MA/O men with normal testosterone (*p* = 0.04, Figure 1E). There were no differences among groups in carbohydrate state 3, 3S, or state 4 respiration, or respiratory acceptor control ratio (RCR) (Figure 1B–D,G).

**FIGURE 2:**
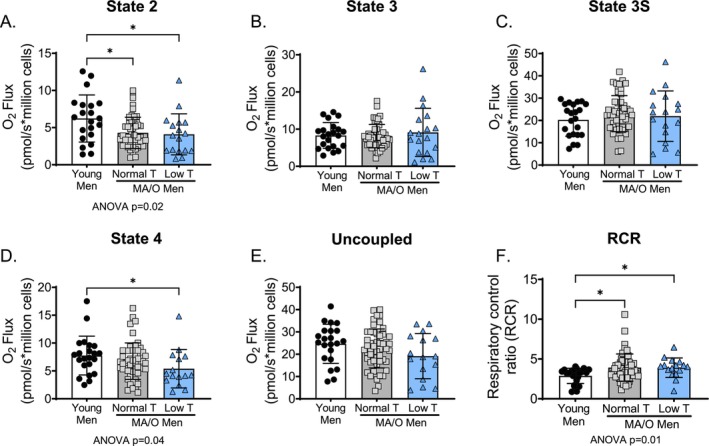
Lipid supported mitochondrial function in human peripheral blood mononuclear cells (PBMCs). (A) State 2 (leak state), (B) State 3 (CI coupled), (C) State 3S (CI + CII coupled), (D) State 4 (leak respiration) respiration, (E) uncoupled respiration (maximal respiration), (F) Respiratory acceptor control ratio (RCR; State 3S/State 4), in young and middle aged/older (MA/O) males with normal testosterone and low testosterone. *p* values represent a one‐way ANOVA, with Tukey post hoc or Kruskal–Wallis with Dunn's multiple comparison if data were not normally distributed. *p < 0.05, **p < 0.01 (*n* = 15–33/group). Data are mean ± SD.

Lipid‐supported state 2 respiration was lower (Figure 2A; both *p* < 0.05) and lipid‐supported RCR was greater (Figure 2F; both *p* < 0.05) in MA/O men compared to young men, regardless of gonadal status. Lipid‐supported state 4 respiration was lower in MA/O men with low testosterone compared to young men (*p* = 0.04; Figure 2D). Lipid‐supported state 3, 3S, and uncoupled respiration were not different across groups (Figure 2B,C,E).

**FIGURE 3:**
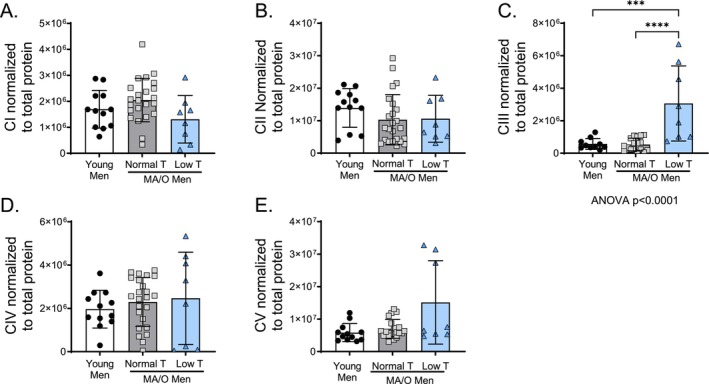
Mitochondrial complex protein abundance in isolated human peripheral blood mononuclear cells (PBMCs). Mitochondrial proteins (A) complex I (CI), (B) complex II (CII), (C) complex III (CIII), (D) complex IV (CIV), and (E) complex V (CV) in young and middle aged/older (MA/O) males with normal testosterone and with low testosterone. *p* values represent a one‐way ANOVA, with Tukey post hoc or Kruskal–Wallis with Dunn's multiple comparison if data were not normally distributed. *p < 0.05, **p < 0.01 (*n* = 15–33/group). Means ± SD.

The effects of age and testosterone status on mitochondrial complex I‐V abundance from isolated PBMCs are presented in Figure 3A–E. There were no differences in mitochondrial complex I, II, IV, and V protein abundance across groups; however, MA/O men with low testosterone had higher mitochondrial complex (CIII) compared to young (*p* = 0.0002) and MA/O men with normal testosterone (*p* < 0.0001; Figure 3C).

### 3.3 The Effects of Age and Gonadal Status on SIRT1 and SIRT3 Abundance

**FIGURE 4:**
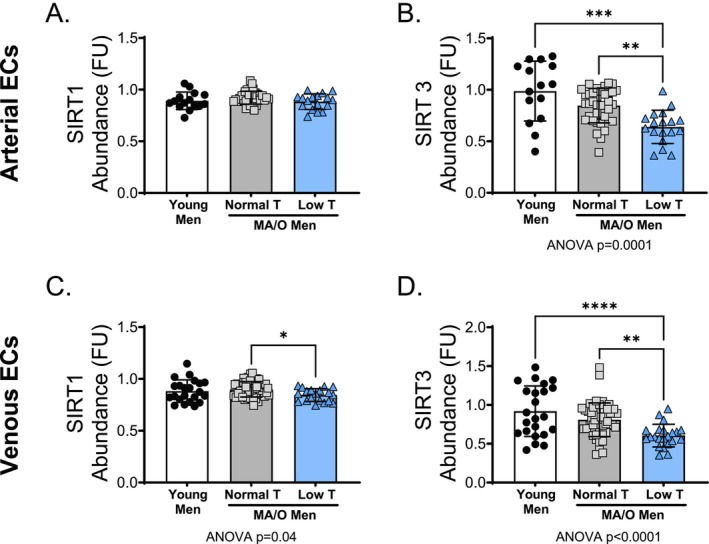
SIRT1 and SIRT3 abundance in human endothelial cells (ECs). (A) SIRT1 and (B) SIRT3 abundance in arterial endothelial cells and (C) SIRT1 and (D) SIRT3 abundance in peripheral venous endothelial cells in young and middle aged/older (MA/O) males with normal testosterone and with low testosterone. *p* values represent a one‐way ANOVA, with Tukey post hoc or Kruskal–Wallis with Dunn's multiple comparison if data were not normally distributed. *p < 0.05, **p < 0.01 (*n* = 15–33/group). Means ± SD.

The effects of age and testosterone status on SIRT1 and SIRT3 abundance were determined in arterial and venous endothelial cells. Arterial and venous endothelial cell SIRT3 abundance was lower in MA/O men with low testosterone compared to young (*p* = 0.0001) and MA/O men with normal testosterone (*p* = 0.003) (Figure 4B,D; all *p* = 0.003–*p* < 0.001). There were no differences in arterial endothelial cell SIRT1 abundance across groups (Figure 4A); however, venous endothelial cell SIRT1 abundance was lower in MA/O men with low testosterone compared to MA/O with normal testosterone (*p* = 0.03; Figure 4C and Supplemental Figure S2,S3).

### 3.4 Effects of Age and Gonadal Status on Mitochondrial Fission and Fusion

**FIGURE 5:**
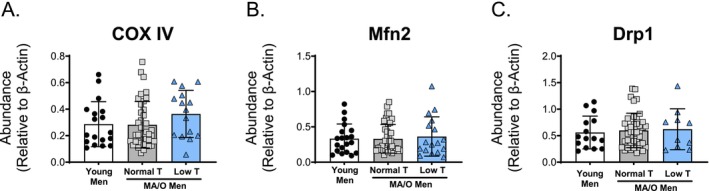
Mitochondrial fission and fusion protein abundance in isolated human peripheral blood mononuclear cells (PBMCs). Protein abundance of markers of mitochondrial fission and fusion including: (A) COX IV, (B) Mfn2, and (C) Drp1 in young and middle aged/older (MA/O) males with normal testosterone and with low testosterone. *p* values represent a one‐way ANOVA, with Tukey post hoc or Kruskal–Wallis with Dunn's multiple comparison if data were not normally distributed. *p < 0.05, **p < 0.01 (*n* = 15–33/group). Means ± SD.

To further elucidate the effects of age and testosterone status on mitochondrial dynamics, key mitochondrial fission and fusion signaling proteins from isolated PBMCs were measured. There were no differences in COX IV, Mfn2, or Drp1 protein abundance across groups (Figure 5A–C).

### 3.5 Effects of Age and Gonadal Status on Biomarkers of Endothelial Cell Redox Balance

**FIGURE 6:**
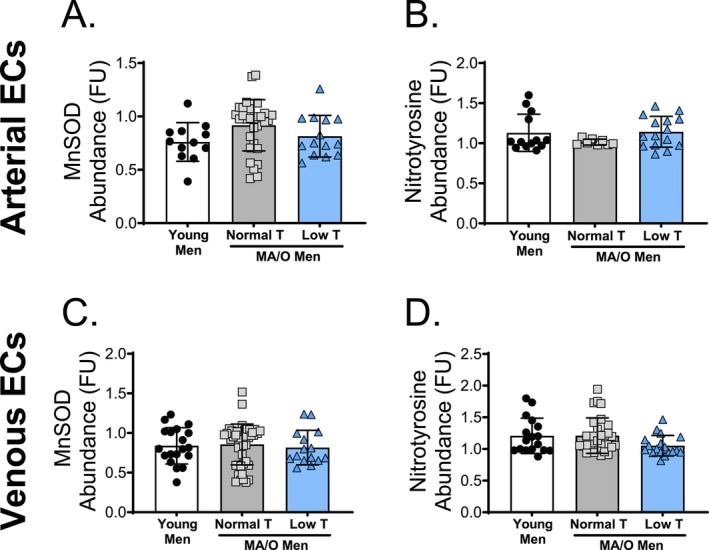
Biomarkers of Redox Balance in human endothelial cells (ECs). Immunofluorescence staining of biopsied ECs of (A) arterial MnSOD, (B) arterial nitrotyrosine, (C) venous MnSOD, (D) venous nitrotyrosine, in young and middle aged/older (MA/O) males with normal testosterone and with low testosterone. *p* values represent a one‐way ANOVA, with Tukey post hoc or Kruskal–Wallis with Dunn's multiple comparison if data were not normally distributed. *p < 0.05, **p < 0.01 (*n* = 15–33/group). Means ± SD.

The effects of age and testosterone status on proteins implicated in redox balance in endothelial cells were determined. There were no differences in arterial or venous endothelial cell expression of MnSOD (Figure 6A,C and Supplemental Figure  S4) or nitrotyrosine across groups (Figure 6B,D).

### 3.6 Correlation Analyses

Serum testosterone levels were directly correlated with FMD (*R* = 0.27; *p* = 0.008), suggesting that higher serum testosterone is associated with greater FMD. Serum testosterone level was negatively correlated with COX IV (*p* = 0.02, Table 2). Free testosterone levels were negatively associated with arterial MnSOD (*p* = 0.04), whereas estradiol levels were positively correlated with arterial MnSOD (*p* = 0.02), but not with venous MnSOD (*p* = 0.15, Table 2). Estradiol levels were negatively correlated with arterial SIRT3 abundance (*p* = 0.024), and venous SIRT3 abundance (*p* = 0.05).

**TABLE 2:** Spearman correlations.

Variables	Testosterone		Free testosterone		Estradiol		SHBG		FSH		LH		SBP		BMI	
	*r*	*p*	*r*	*p*	*r*	*p*	*r*	*p*	*r*	*p*	*r*	*p*	*r*	*p*	*r*	*p*
FMD	**0.27**	**0.008**	−0.005	0.96	−0.131	0.20	0.072	0.483	−0.136	0.188	0.133	0.196	**−0.39**	**< 0.001**	**−0.39**	**< 0.001**
Carb RCR	−0.03	0.80	0.02	0.838	0.037	0.714	−0.057	0.609	−0.049	0.660	−0.124	0.259	−0.06	0.64	0.17	0.12
Lipid RCR	−0.13	0.25	0.03	0.788	0.104	0.364	0.123	0.268	−0.012	0.912	−0.047	0.673	0.018	0.87	0.20	0.08
Arterial SIRT1	−0.18	0.12	−0.15	0.233	0.217	0.069	0.140	0.246	0.043	0.721	−0.088	0.468	0.08	0.50	0.06	0.64
Venous SIRT1	0.03	0.75	−0.09	0.425	0.107	0.318	0.082	0.448	−0.001	0.993	−0.052	0.630	−0.11	0.29	0.00	0.96
Arterial SIRT3	0.19	0.17	0.126	0.368	**−0.306**	**0.024**	0.099	0.480	−0.074	0.596	−0.093	0.506	−0.11	0.45	−0.20	0.14
Venous SIRT3	0.12	0.33	0.109	0.361	−0.225	0.054	−0.015	0.902	−0.098	0.411	−0.125	0.292	−0.17	0.15	−0.06	0.59
COX IV	**−0.24**	**0.02**	0.092	0.404	−0.103	0.343	0.023	0.835	0.042	0.699	−0.084	0.442	0.02	0.88	−0.00	0.99
MFN2	−0.09	0.40	−0.043	0.699	0.047	0.667	0.049	0.655	−0.074	0.499	−0.109	0.317	0.04	0.71	0.11	0.34
DRP1	−0.21	0.10	−0.013	0.917	−0.043	0.727	−0.040	0.745	−0.008	0.949	0.120	0.331	−0.12	0.32	0.12	0.34
Arterial MnSOD	0.12	0.37	**−0.285**	**0.037**	**0.313**	**0.019**	0.253	0.063	0.007	0.960	0.072	0.602	−0.15	0.28	0.18	0.19
Venous MnSOD	0.04	0.75	−0.139	0.221	0.155	0.167	0.102	0.369	0.078	0.490	0.166	0.141	−0.14	0.22	0.04	0.76

*Note:* Bold font indicates significant correlation.

Abbreviations: BMI, body mass index; COX IV, cytochrome c subunit 4; DRP1, dynamin‐related protein 1; FMD, flow mediated dilation; FSH, follicle stimulating hormone; LH, luteinizing hormone; MFN2, mitofusin 2; mnSOD, superoxide dismutase 2; RCR, respiratory control ratio; SBP, systolic blood pressure; SIRT1, sirtuin 1; SIRT3, sirtuin 3.

To examine the potential influence of testosterone status on outcomes of interest independent of age, correlations were determined among MA/O men only. Serum testosterone levels were negatively correlated with COXIV (*p* = 0.03, Table 3). Estradiol levels remained inversely correlated with arterial SIRT3 abundance (*p* = 0.04), but not venous SIRT3 abundance, and were negatively correlated with COXIV (*p* = 0.04). SHBG was positively correlated with FMD (*p* = 0.02). Interestingly, LH was negatively correlated with carbohydrate RCR (*p* = 0.04).

**TABLE 3:** Spearman correlations in pooled middle‐aged/older men with normal or low testosterone.

Variables	Testosterone		Free testosterone		Estradiol		SHBG		FSH		LH		SBP		BMI	
	*r*	*p*	*r*	*p*	*r*	*p*	*r*	*p*	*r*	*p*	*r*	*p*	*r*	*p*	*r*	*p*
FMD	0.18	0.11	0.07	0.55	0.02	0.87	**0.28**	**0.02**	−0.01	0.96	0.16	0.17	**−0.30**	**0.009**	−0.17	0.14
Carb RCR	−0.001	0.99	0.14	0.30	−0.04	0.89	**−0.25**	**0.05**	−0.07	061	**−0.26**	**0.04**	−0.08	0.56	0.12	0.37
Lipid RCR	−0.10	0.46	0.13	0.33	0.02	0.77	−0.06	0.6	−0.10	0.42	−0.09	0.47	−0.07	0.59	−0.00	0.99
Arterial SIRT1	−0.02	0.90	−0.02	0.88	0.13	0.36	0.11	0.47	0.09	0.55	0.03	0.83	0.05	0.74	−0.06	0.67
Venous SIRT1	0.15	0.21	0.04	0.77	0.06	0.65	0.11	0.40	0.02	0.90	0.01	0.93	−0.16	0.20	−0.05	0.71
Arterial SIRT3	0.25	0.12	0.04	0.80	**−0.32**	**0.04**	0.15	0.35	0.05	0.77	−0.06	0.72	−0.09	0.57	−0.28	0.08
Venous SIRT3	0.24	0.09	0.13	0.93	−0.22	0.10	0.13	0.35	−0.10	0.45	−0.03	0.86	−0.13	0.35	−0.13	0.36
COX IV	**−0.26**	**0.03**	0.21	0.09	**−0.25**	**0.04**	0.07	0.58	−0.04	0.77	−0.12	0.34	−0.01	0.90	−0.04	0.74
MFN2	−0.02	0.85	0.08	0.53	0.06	0.63	0.01	0.92	−0.11	0.38	−0.10	0.40	0.06	0.61	0.12	0.34
DRP1	−0.24	0.08	0.01	0.97	−0.10	0.46	−0.01	0.92	−0.11	0.43	0.12	0.39	−0.17	0.22	0.16	0.27
Arterial MnSOD	0.21	0.17	−0.17	0.29	0.028	0.07	0.21	0.17	−0.01	0.94	0.01	0.93	−0.28	0.07	0.16	0.31
Venous MnSOD	0.05	0.71	−0.15	0.26	0.15	0.24	0.12	0.36	0.03	0.83	0.12	0.35	−0.03	0.80	0.02	0.85

*Note:* Bold font indicates significant correlation.

Abbreviations: BMI, body mass index; COX IV, cytochrome c subunit 4; DRP1, dynamin‐related protein 1; FMD, flow mediated dilation; FSH, follicle stimulating hormone; LH, luteinizing hormone; MFN2, mitofusin 2; mnSOD, superoxide dismutase 2; RCR, respiratory control ratio; SBP, systolic blood pressure; SIRT1, sirtuin 1; SIRT3, sirtuin 3.

FMD was also negatively correlated with SBP (*r* = −0.39, *p* < 0.001) and BMI (*r* = −0.39, *p* < 0.001) in all men, but only negatively associated with SBP in MA/O men (−0.30, *p* = 0.009). SBP and BMI were not significantly associated with any other variables in Tables 2 and 3.

## 4 Discussion

Mitochondrial dysfunction is a hallmark of aging and excessive mitochondrial ROS has been broadly implicated in age‐related vascular endothelial dysfunction (Rossman et al. 2018). However, to our knowledge, the role of mitochondrial function in accelerated vascular aging in MA/O men with low testosterone had not been investigated. The main overarching novel findings from the series of studies conducted here are the important dynamic relations of aging and testosterone levels in healthy men. Our results demonstrated that mitochondrial function in PBMCs, as measured by high resolution respirometry, was altered in MA/O men with low testosterone, as indicated by differences in state 2, 4, uncoupled, and RCR for carbohydrate and lipids. Additionally, endothelial SIRT3 protein abundance was lower in the MA/O men with low testosterone compared to young and MA/O men with normal testosterone, suggesting that MA/O men with low testosterone had mitochondrial dysfunction and reductions in key signaling proteins that regulate mitochondrial biogenesis, mitochondrial ROS and stress response. These findings suggest that interventions aimed at improving mitochondrial health in MA/O men with low testosterone may be a potential therapeutic strategy to reduce or prevent accelerated vascular endothelial dysfunction, thus reducing the risk of CVD and CV‐related mortality in these men.

### 4.1 Vascular Endothelial Function in Middle‐Aged/Older Men With Low Testosterone

We previously demonstrated that MA/O men with low testosterone have greater age‐associated vascular endothelial dysfunction compared to age‐matched peers with normal testosterone. The greater level of vascular endothelial dysfunction was mediated, in part, by oxidative stress, as indicated by an improvement in brachial artery FMD in response to acute vitamin C infusion in the MA/O men with low testosterone but not age‐matched men with normal testosterone (Babcock et al. 2022). These findings provided the scientific premise for determining the potential cellular/molecular pathways that contribute to oxidative stress and vascular endothelial dysfunction in MA/O men with low testosterone.

Although the unadjusted mean FMD level was not statistically different between MA/O men with normal and low testosterone, the mean difference between the groups (~1.1%) is considered clinically significant as reductions in FMD of 1% are associated with a ~9%–13% increase in CVD risk (Inaba et al. 2010; Green et al. 2011). Additionally, we believe that the associations between FMD and testosterone were weaker in the present study compared to our previously published observations because in that study, MA/O men were matched for BMI and SBP (Babcock et al. 2022). In the present study, when we adjust for BMI and SBP, FMD is significantly lower in MA/O men with low testosterone compared to young and MA/O men with normal testosterone, consistent with our previous observations (Supplemental Figure S1) (Babcock et al. 2022).

### 4.2 Accelerated Vascular Aging in Middle‐Aged/Older Men With Low Testosterone: Implications of Mitochondrial Health

Mitochondria regulate ATP production through oxidative phosphorylation to support the energy demands of endothelial cells to conduct the physiological processes involved in endothelial function (Tang et al. 2014). However, endothelial cells have a relatively lower energy demand than other cell types and produce ATP primarily through glycolysis (Pang et al. 2024; Grossini et al. 2025). In addition to meeting the energy demands of endothelial cells, mitochondria also play an important role in mediating moderate levels of ROS production for normal physiological signaling that could alter adhesion molecules, induce angiogenesis and vascular smooth muscle cell growth, and regulate vascular tone (Taniyama and Griendling 2003). Mitochondria are particularly prone to damage from excess ROS and are also a major source of ROS (Palma et al. 2024). This detrimental cycle of ROS‐induced ROS production from mitochondria is a major contributor to mitochondrial dysfunction (Zorov et al. 2014).

We found that PBMC mitochondrial function, in the presence of carbohydrate substrate utilization, was reduced in MA/O men with low testosterone compared to young men with normal testosterone, which may have contributed to their lower maximal respiration (uncoupled respiration). Glycolysis is the primary substrate to produce ATP for endothelial cell mitochondria; however, mitochondria can also utilize lipids to produce ATP through beta‐oxidation (Y. Wang et al. 2019). Similar to carbohydrates, lipid‐supported state 2 respiration was lower in MA/O men with low testosterone relative to young men. In addition, state 4 respiration was lower; however, the lipid‐supported RCR was greater in MA/O men with low testosterone compared to young men. The lower state 2 and state 4 respiration suggests greater proton leak and impaired mitochondrial proton gradient when utilizing lipid substrate as the primary fuel source, thus negatively impacting maximal mitochondrial respiration. However, uncoupled respiration did not differ across the groups, and RCR was greater in MA/O men with lower testosterone compared to young men when lipids were utilized. The higher lipid‐supported RCR in both groups of MA/O men, regardless of testosterone status, in comparison to young men was unexpected, as higher RCR generally indicates a more efficient oxidative phosphorylation (Martin 2011). It is possible that the mitochondria in MA/O men have shifted their primary fuel source from carbohydrates to lipids as a compensatory mechanism to overcome impaired maximal mitochondrial respiration in the presence of carbohydrates. Considering ROS can be both pathophysiological in excess and beneficial in homeostasis for vascular and mitochondrial function, future studies are warranted to determine how ROS may impact mitochondrial substrate utilization in endothelial cells specifically (Zinkevich and Gutterman 2011; Clempus and Griendling 2006; Starkov 2008).

Within the vasculature, ROS is important for intracellular signaling that can regulate vascular constriction, dilation, and remodeling (Xu and Touyz 2006). During pathological stressors such as oxidative stress, inflammation, and mitochondrial dysfunction, excess ROS can lead to greater or enhanced beta‐oxidation (Schönfeld and Reiser 2017; Jørgensen et al. 2021; Hansen et al. 2021). Thus, various stressors may influence mitochondrial substrate utilization and the shift in the substrate utilization may serve as a compensatory mechanism to maintain energy demand (Cortassa et al. 2019). Interestingly, with advancing age, skeletal muscle mitochondria have marked inability to switch from lipid to glucose metabolism. Thus, lipid metabolism may serve as the primary mechanism of fuel utilization due to impaired glucose metabolism with aging (Petersen et al. 2015). Furthermore, preclinical evidence suggests that high lipid substrate utilization from microvascular endothelial cell mitochondria can induce mitochondrial dysfunction (Hansen et al. 2021). Given that the PBMC mitochondrial RCR from MA/O men with low testosterone was higher during lipid supported respiration but not significantly different between groups during carbohydrate substrate utilization, it is plausible that these men relied on lipid metabolism as a compensatory mechanism to maintain energy demands, suggesting overall less substrate flexibility. In contrast, MA/O men with low testosterone had higher circulating triglycerides, arguing in favor of appropriate substrate flexibility. Future studies are needed to further evaluate the possible mechanisms associated with the compensatory mitochondrial phenotype observed in these studies and the role of beta‐oxidation‐induced mitochondrial dysfunction through the lens of the vasculature in aging men.

### 4.3 Sirtuin Protein Abundance in Middle‐Aged/Older Men With Low Testosterone

SIRTs are a conserved protein family of the class III histone deacetylases that have been implicated in longevity and prevention of age‐associated diseases; however, the underlying mechanisms of SIRTs in altering the biological aging process remain unclear. To date, seven mammalian SIRTs have been identified, all of which have varying biological processes and are distributed into different subcellular compartments including the nucleus, cytoplasm, and mitochondria (Cencioni et al. 2015; Min et al. 2001). Among the SIRT family, SIRT1 and SIRT3 are particularly instrumental in regulating vascular function, and maintaining mitochondrial homeostasis, respectively (Cencioni et al. 2015; Cheng et al. 2025; Donato et al. 2011).

SIRT1 is thought to be the most impactful modulator of vascular function and is highly expressed in endothelial cells (D'Onofrio et al. 2015; Potente et al. 2007; Sanchez‐Fidalgo et al. 2012). SIRT1 can directly promote endothelium‐dependent dilation via activation of endothelial nitric oxide synthase (Mattagajasingh et al. 2007). Furthermore, SIRT1 can modulate vascular morphology through regeneration of endothelial cells and regulation of vascular adhesion proteins (Man et al. 2019). SIRT1 also plays an important role in combating pathophysiological conditions associated with oxidative stress by shuttling between the endothelial cell nucleus and cytoplasm to initiate protective mechanisms against oxidative stress‐induced endothelial cell dysfunction through cellular proliferation, growth, and inhibition of cell death pathways (Hou et al. 2010). Our results demonstrated that MA/O men with low testosterone had lower venous endothelial cell SIRT1 abundance compared to age‐matched men with higher testosterone, which may contribute to the accelerated vascular aging observed in MA/O men with low testosterone. While lower SIRT1 in the venous and not arterial cells is perplexing, it could be related to differences in blood pressure and hemodynamics (Ballermann et al. 1998; Lu and Kassab 2011), although this hypothesis warrants further investigation.

SIRT3 is primarily a mitochondrial enzyme that plays an important role in mitochondrial function and can protect against oxidative stress (Nogueiras et al. 2012; He et al. 2019; Dikalova et al. 2020). SIRT3 can regulate various aspects of mitochondrial biology from mitochondrial antioxidant defenses, ATP generation, and mitochondrial fission and fusion (McDonnell et al. 2015; Tao et al. 2010). With advancing age, cardiac and vascular endothelial cell SIRT3 expression declines and contributes to age‐related CVD risk (Koentges et al. 2016; He et al. 2019). Although reduced SIRT3 abundance has been implicated in accelerated vascular aging, the underlying mechanisms mediating this response remain unclear. Here, we demonstrated for the first time that arterial and venous endothelial cells from MA/O men with low testosterone had lower SIRT3 abundance than both young men and MA/O men with normal testosterone. Although we did not observe differences in MnSOD or nitrotyrosine expression, it is possible that the lower SIRT3 expression contributed to reduced abundance of other antioxidant enzymes, ROS and oxidative damage. Furthermore, while we did not observe any changes to mitochondrial fission and fusion proteins (i.e., COX IV, Mfn2, and Drp1), mitochondrial complex III abundance was greater in MA/O men with low testosterone compared to young and MA/O men with normal testosterone. This observation was notable because complex III is essential for transferring electrons to cytochrome c, which subsequently delivers electrons to complex IV for oxygen reduction and ATP production (Chandel 2010). Therefore, it is possible that the observed higher levels of complex III within mitochondria of MA/O men with low testosterone explain the observed greater RCR in these men. However, the specific underlying role of SIRT3 in mitochondrial dysfunction associated with MA/O men with low testosterone remains to be fully elucidated.

Collectively, the lower endothelial cell abundance of SIRT1 and SIRT3 protein in MA/O men with low testosterone, relative to young and MA/O men with normal testosterone, provides insight into the potential mechanisms of accelerated vascular aging and the observed mitochondrial dysfunction in MA/O men with low testosterone. These novel findings provide further support for the growing interest in SIRTs as a potential target in anti‐aging medicine and a key regulator of vascular aging. However, additional work is needed to determine the specific mechanistic role in which SIRT1 and SIRT3 mediate accelerated vascular aging in MA/O men with low testosterone.

### 4.4 Association of Estradiol and Accelerated Vascular Aging in Middle‐Aged/Older Men With Low Testosterone

Testosterone is aromatized to estradiol and therefore we examined whether testosterone via effects of estradiol was associated with select mechanisms highlighted in the present study. Low levels of estradiol have been implicated in vascular dysfunction and elevated CVD risk in men and women (SenthilKumar et al. 2023). In hypogonadal older men, low‐dose estrogen supplementation can improve vascular function (Komesaroff et al. 2001). Estradiol has been shown to have direct antioxidant properties and up‐regulate the expression of important endogenous antioxidant enzymes, including MnSOD, to protect mitochondria from oxidative damage (Borrás et al. 2010). In this regard, the results from the present study show that higher estradiol levels were correlated with higher MnSOD. Interestingly, estradiol was inversely related with SIRT3 abundance in the pooled cohort of MA/O men. These data suggest that age and testosterone status in MA/O men may influence mitochondrial health possibly through estradiol and SIRT3 signaling. Thus, future studies are needed to delineate how sex hormones, as well as other gonadal hormones affect mitochondrial function and vascular aging.

### 4.5 Limitations and Experimental Considerations

We acknowledge that the present study has several important limitations. First, there is large variability in the mitochondrial respirometry measures in PBMCs. Potential variability could be due, but not limited to, immune cell composition (i.e., lymphocytes vs. monocytes), as well as other lifestyle factors. Additionally, given that PBMCs are in circulation, it is plausible that ROS generated from PBMCs is transferred to endothelial cells. In a sub‐population of men in the present study, we measured ROS production in PBMCs using high‐resolution respirometry (O2K‐FluoRespirometer) via Amplex UltraRed assay to detect hydrogen peroxide release while simultaneously measuring mitochondrial respiration but the differences between the groups were not statistically different (data not shown). Whether PBMC generated ROS mediates effects in endothelial cells requires further investigation. Future studies should also consider measuring nicotinamide adenine dinucleotide (NAD) levels given the relation of NAD with aging. Additionally, because sirtuins require NAD to function, NAD levels could be contributing to the observed differences in sirtuins, however this hypothesis requires further investigation. We recognize that only a limited number of endothelial cell proteins were assessed in the present study, and that proteins involved in modulation of endothelial function, including the androgen receptor were not included. Notably, the total number of proteins that can be assessed with the endothelial cell biopsy technique is limited; thus, we selected to measure proteins that are highly implicated in mitochondrial function and ROS. The selected proteins enabled us, for the first time, to characterize the impact of gonadal status on vascular function in healthy humans and the influence of testosterone in the absence of confounding clinical disease.

## 5 Conclusions

Middle‐aged/older men with low testosterone levels have greater vascular endothelial dysfunction than aged‐matched men with normal testosterone levels, which contributes to increased CVD risk and mortality. Therefore, understanding the mechanisms by which low testosterone in MA/O men mediate accelerated vascular aging would provide important insights into potential therapeutic approaches. For the first time, we provided evidence that mitochondrial health may play an important role in accelerated vascular aging in MA/O men with low testosterone. Moreover, this study identified SIRTs as putative modulators of mitochondrial dysfunction in MA/O men with low testosterone. Importantly, mounting evidence suggests that targeting SIRTs in various age‐related diseases, such as CVD, could be an effective therapeutic strategy. Thus, future studies are warranted to investigate whether targeting SIRTs can mitigate the accelerated vascular aging in men with low testosterone.

## Author Contributions

K.L.M. conceived and designed the research. K.L.H. and B.L.S. provided medical oversight of the study participants, evaluated inclusion and exclusion criteria, and reviewed adverse events. K.L.M. M.C.B. and L.E.D. collected the data. B.L.N, M.N.K, Z.S.C., and K.L.M. analyzed and performed the statistical analysis of the data, and drafting of the manuscript. All authors helped in the interpretation of the data and approving of the final version of the manuscript.

## Funding

This research was supported by National Institutes of Health R01AG049762, National Institutes of Health U54AG062319, National Institutes of Health T32AG000279, Colorado Clinical and Translational Sciences Institute UL1 TR001082, National Institutes of Health P30 DK048520, and Veteran Affairs Eastern Colorado GRECC.

## Conflicts of Interest

The authors declare no conflicts of interest.

## Supporting information


**Data S1:** acel70457‐sup‐0001‐Supinfo.pdf.

## Data Availability

The data that support the findings of this study are available from the corresponding author upon reasonable request.
