# Supplementary material for: Endothelial Sirtuins and Mitochondrial Function Are Associated With Testosterone Status: Implications for Accelerated Vascular Aging in Middle‐Age and Older Men With Low Testosterone
Source: Aging Cell. 2026 Apr 15;25(4):e70457. doi: 10.1111/acel.70457 (PMC13083223; doi:10.1111/acel.70457)
Supplement: Supplementary file 1 — Data S1: acel70457‐sup‐0001‐Supinfo.pdf. [file ACEL-25-e70457-s001.pdf]

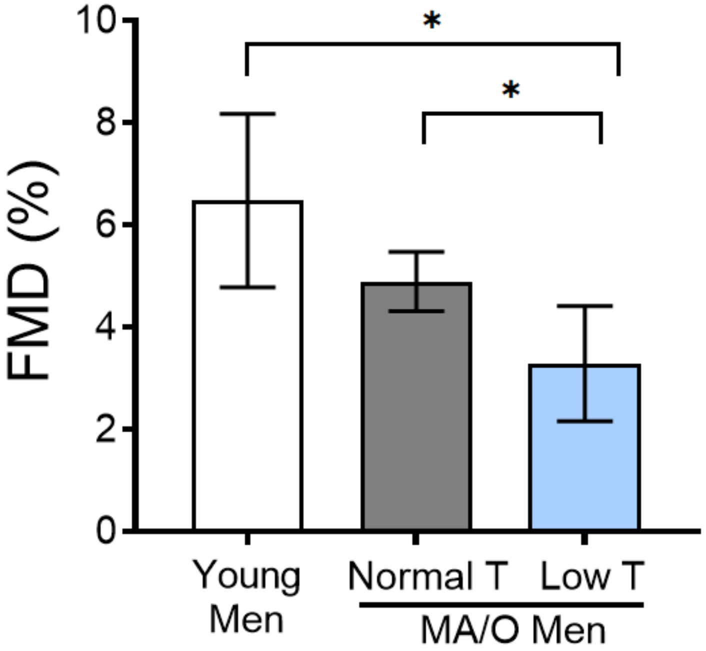

Supplemental Figure 1. Adjusted mean FMD in young and middle-age and older men with normal testosterone and in middle-age and older men with low testosterone. Means adjusted for systolic blood pressure and body mass index. Data are means with upper and lower 95% confidence intervals. * p=0.04.

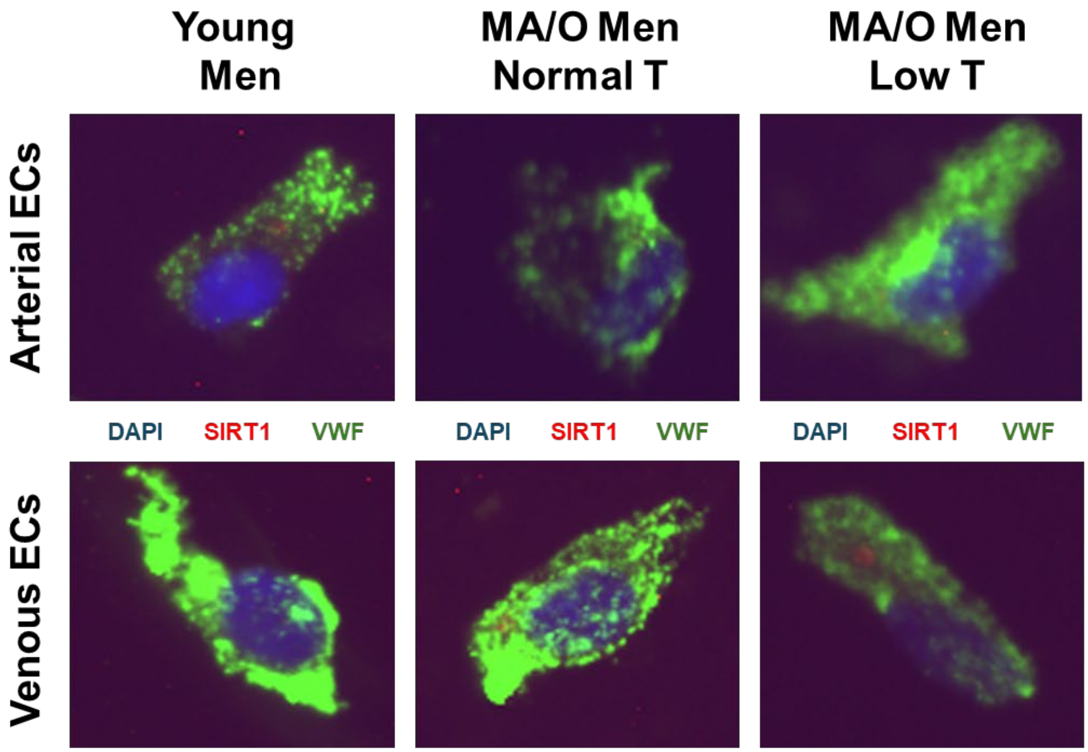

Supplemental Figure 2. Representative immunofluorescent staining for SIRT1 in Arterial and Venous Endothelial Cells (ECs) from Young Men, Middle Aged/Older (MA/O) Men with Normal Testosterone (T), and Middle Aged/Older Men with Low Testosterone.

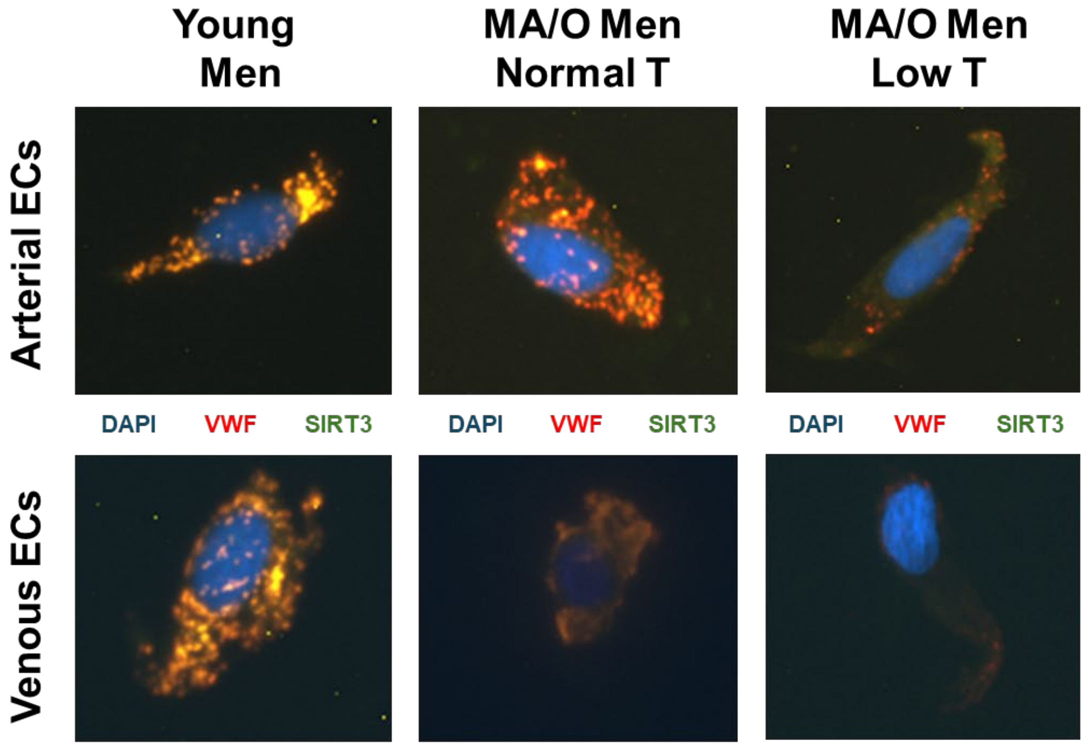

Supplemental Figure 3. Representative immunofluorescent staining for SIRT3 in Arterial and Venous Endothelial Cells (ECs) from Young Men, Middle Aged/Older (MA/O) Men with Normal Testosterone (T), and Middle Aged/Older Men with Low Testosterone.

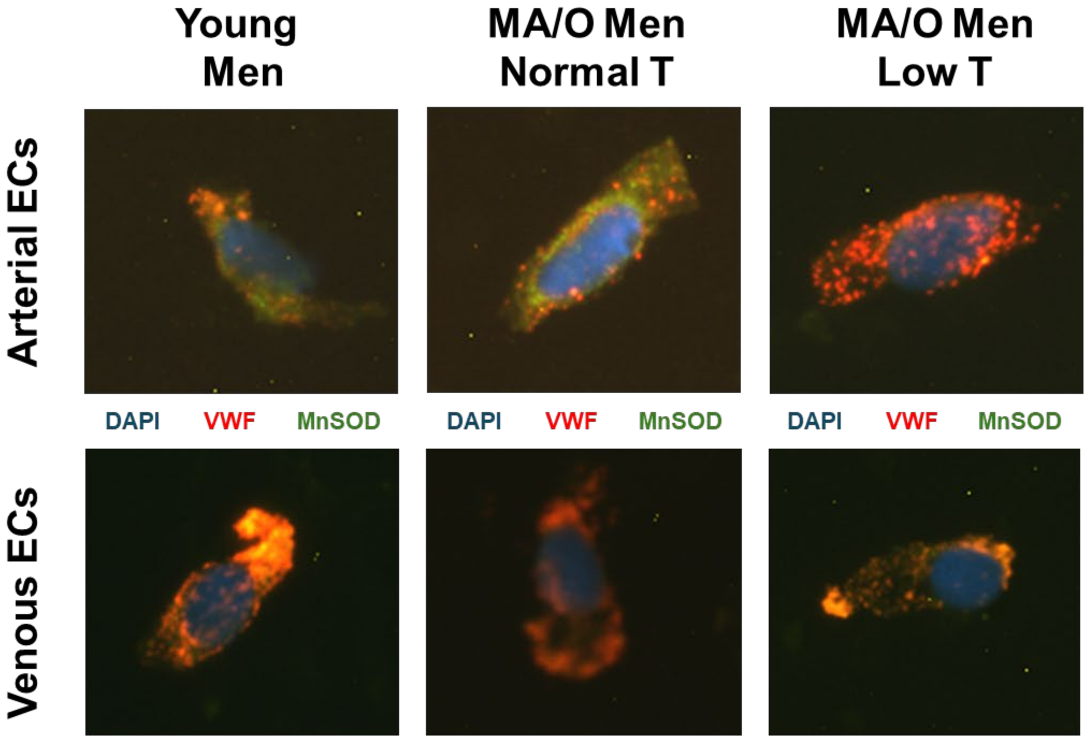

Supplemental Figure 4. Representative immunofluorescent staining for MnSOD in Arterial and Venous Endothelial Cells (ECs) from Young Men, Middle Aged/Older (MA/O) Men with Normal Testosterone (T), and Middle Aged/Older Men with Low Testosterone.
